# To Prevent Ophthalmia Neonatiorum

**Published:** 1908-05

**Authors:** 


### TO PREVENT OPHTHALMIA NEONATORUM.

The first conviction under the law recently passed in Baltimore to prevent infants from becoming blind was obtained in the criminal court March 27. Mrs. Mary Fogler, a midwife, was found guilty of violating the law and was fined $25.00 and cost, amounting to $43.00.

The law in question provides that if at any time within two weeks after the birth of an infant one or both of its eyes or eyelids be reddened or inflamed, the nurse or person in charge of the infant shall refrain from the application of any remedy, and shall report immediately such condition to the Health Commissioner or a qualified physician. The penalty for violating the law is a fine not exceeding $100, or imprisonment in jail for not more than six months, or both fine and imprisonment.

It was shown by the testimony that Mrs. Fogler attended Mrs. Mary Conway, 1610 Burroughs street, whose child was born February 6 last, and the infant had lost the sight of one eye. Mrs. Conway testified that when the infant's eyes became affected Mrs. Fogler told her not to get a doctor, and the case was not reported to any physician. Finally the child was taken to a hospital and the prosecution of Mrs. Fogler followed.

According to Mrs. Fogler's own statement, Judge Wright said, in deciding the case, she had violated the law by not reporting the case to a physician. Her want of knowledge, or refusal to obey the law, the Judge added, had caused the child to become blind in one eye. As it was the first case of the kind to be tried, the maximum penalty was not imposed.

There are few diseases in which disaster is more likely to follow delay in treatment than in gonorrhoeal ophthalmia, and the publicity given to such cases will be of great value in educating mothers as to the danger of sore eyes in an infant, and to the necessity of a careful examination at the incipiency of all such conditions.

